# An Eight Year Experience of Autologous Oocyte Vitrification for Infertile Patients Owing to Unavailability of Sperm on Oocyte Retrieval Day

**DOI:** 10.3389/fmed.2021.663287

**Published:** 2021-10-26

**Authors:** Xiao Fu, Xiaojie Liu, Jing Li, Meng Zhang, Jingjing Jiang, Qianqian Chen, Mei Li, Shanshan Gao, Jinlong Ma

**Affiliations:** ^1^Cheeloo College of Medicine, Shandong University, Jinan, China; ^2^Center for Reproductive Medicine, Shandong University, Jinan, China; ^3^National Research Centre for Assisted Reproductive Technology and Reproductive Genetics, Shandong University, Jinan, China

**Keywords:** oocyte vitrification, survival rate, live birth, obstetric outcomes, blood lipid level

## Abstract

**Objective:** The objective of this study was to provide a descriptive analysis of the clinical outcomes achieved in oocyte vitrification in cases where sperm was unavailable on oocyte retrieval day, and to identify predictors of oocyte survival.

**Methods:** This retrospective cohort study used data from a university-affiliated reproductive medical center. There were 321 cycles in which some of, or all oocytes were vitrified owing to the unavailability of sperm between March 2009 and October 2017. A descriptive analysis of the clinical outcomes including both fresh embryo transfers and cryopreserved embryo transfers was provided. The ability of an individual parameter to forecast oocyte survival per thawing cycle was assessed by binary logistic regression analysis. The cumulative probability of live birth (CPLB) was estimated by using the Kaplan-Meier method according to the total number of oocytes thawed in consecutive procedures.

**Results:** The average survival rate was 83.13%. High-quality embryo rate and blastocyst rate decreased significantly decreased significantly in vitrification oocyte group compared to fresh control oocytes. The comparison of sibling oocytes in part-oocyte-vitrified cycles shows fewer high-quality embryos developed in the vitrified group. The live birth rate per warmed-oocyte was 4.3%. Reasons for lack of sperm availability on oocyte retrieval day and serum cholesterol levels were found to be associated with oocyte survival rate in the present study. Kaplan-Meier analysis showed no significant difference in CPLB between patients ≤35 vs. >35 years.

**Conclusions:** Oocyte vitrification is an indispensable and effective alternative when sperm are not available on oocyte retrieval day. The present study provided evidence that oocytes from infertile couples were more likely to suffer oocyte/embryo vitrification injury. Clinicians need to take this into account when advising patients in similar situations. Further studies will be necessary to clarify the correlation between serum metabolism parameters and human oocyte survival after vitrification.

## Introduction

Oocyte freezing is no longer considered an experimental method by the American Society for Reproductive Medicine (1). Oocyte vitrification is gradually becoming a useful adjunct to routine *in vitro* fertilization (IVF) in various clinical scenarios such as the unavailability of sperm at the time of egg retrieval (2–4), and for couples who do not wish to cryopreserve supernumerary embryos in cases where plenty of oocytes are retrieved (5). Another indication for oocyte vitrification that has now become a reality is the establishment of donor oocyte banks (6–8). Oocyte cryopreservation for deferring child-bearing and fertility preservation in cancer patients has also entered clinical practice (9–12).

Reports of donor oocyte vitrification have so far been encouraging. In a sibling cohort study of recipient cycles, similar embryo development has been shown from fresh vs. vitrified oocytes (13). Several well-controlled studies involving donor oocytes have shown that clinical outcomes with vitrified oocytes are comparable to those with fresh oocytes (7, 14–16). A large study of a donation program reported by Cobo et al. has demonstrated comparable obstetric and perinatal outcomes from vitrified vs. fresh oocytes (17). These results have confirmed the further application of oocyte vitrification in assisted reproduction treatment for medical indications.

Although oocyte vitrification has been demonstrated as a successful and stable technique in donor programs, these results might provide overly optimistic evidence for oocyte vitrification where there are medical indications in infertile patients. Different oocyte sources may have varying inherent qualities that affect vitrification outcomes (9, 18). However, reports related to autologous oocyte vitrification in infertile patients are few and inconsistent (12, 19). A study of sibling oocytes from 44 patients undergoing IVF showed reduced rates of fertilization and embryo development after oocyte vitrification (19). Another study included 128 autologous vitrified/warmed oocyte cycles from IVF cycles and demonstrated significantly higher implantation rates (43 vs. 35%) and clinical pregnancy (57 vs. 44%) with vitrified-warmed compared to fresh oocytes (12).

This study aims to describe the outcomes we have achieved in our 8-year experience of oocyte vitrification owing to unavailable sperm on oocyte retrieval day. Analyses were performed to find relevant factors regarding oocyte survivability. This relatively large data set adds to the limited information currently available regarding the clinical application of vitrified autologous oocytes for medical indications.

## Materials and Methods

**Figure 1:**
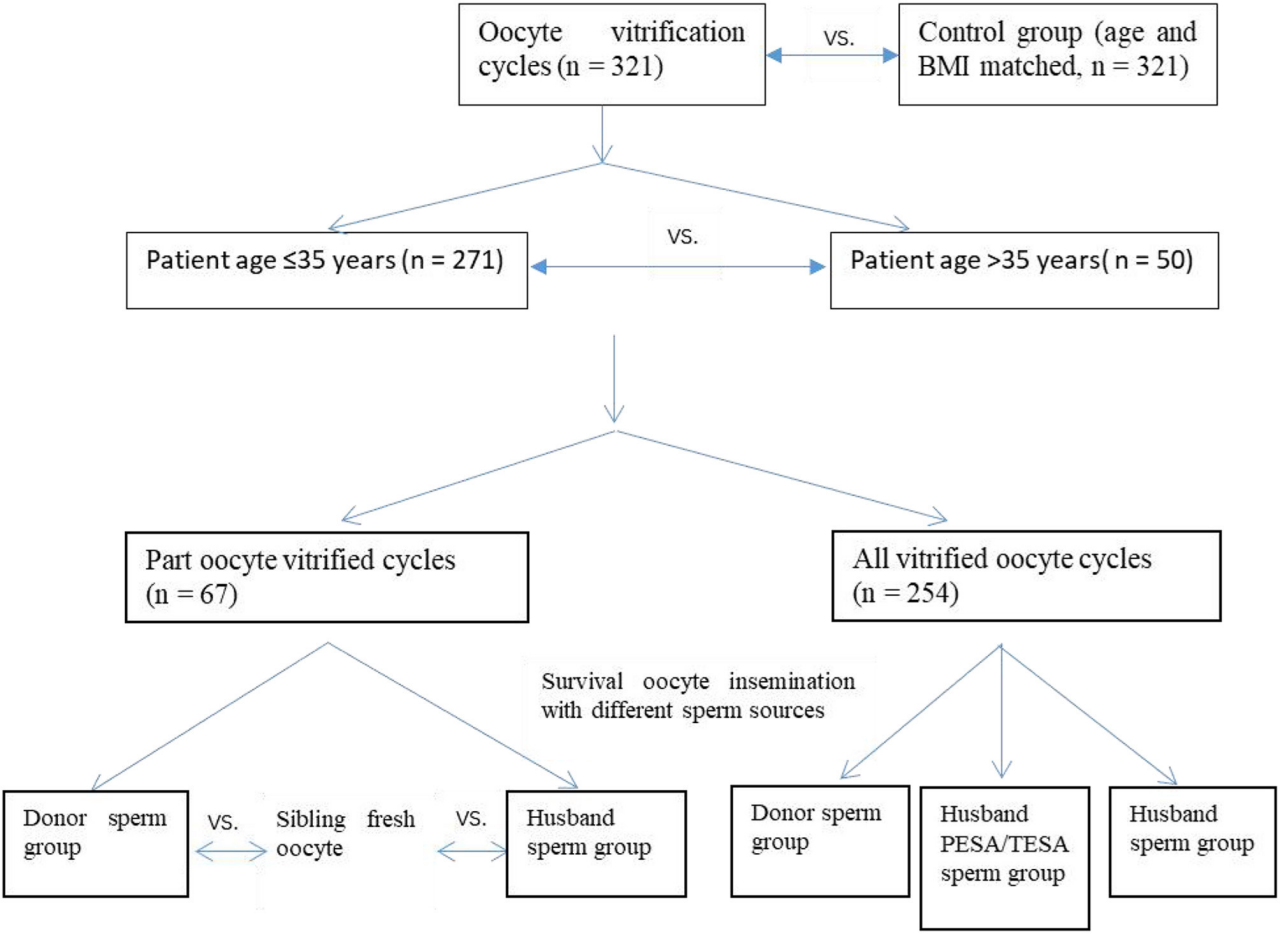
Description of experimental groups and comparisons.

The ethics committee at the Center for Reproductive Medicine, Shandong University approved this clinical application. Couples chose oocyte cryopreservation because of the unavailability of sperm at the time of oocyte retrieval as an alternative to using donor semen. The control group consisted of age and Body Mass Index (BMI)-matched patients, who were undergoing intracytoplasmic sperm injection (ICSI) treatment for male factor infertility (Figure 1).

### Vitrification Kit, Oocyte Vitrification-Warming, and IVF Procedures

This study included two commercially available kits: the MediCult (MC) kit (MediCult Vitrification Cooling, Copenhagen, Denmark) and Kitazato (KT) kit (Kitazato Biopharma Co., Ltd., Shizuoka, Japan), and one Modified kit prepared in our lab. The penetrating cryoprotectants in the MC kit were ethylene glycol (EG) and 1,2-propanediol (PROH). The KT kit included EG and dimethyl sulphoxide (DMSO). The Modified kit was made up of three types of cryoprotectants: ethylene glycol (Sigma-Aldrich, St. Louis, MO, 102466, USA), DMSO (Sigma-Aldrich, St. Louis, MO, D2650, USA), and PROH (Sigma-Aldrich, St. Louis, MO, 544324-068, USA). As for the MC kit and KT kit, equilibration solution included 7.5% EG + 7.5% PROH (DMSO), and vitrification solution included 15% EG + 15% PROH (DMSO) + 0.5 mol/L sucrose, per the instructions. The Modified kit was prepared with M-199 (Gibco Invitrogen Corp., Grand Island, NY, USA) as the basal medium. A 20% serum plasma substitute (SPS) (SAGE, Trumbull, CT, USA) was also added. The equilibration solution for the Modified kit comprised 7.5% EG + 3.75% DMSO + 3.75% PROH, and the vitrification solution comprised 15% EG + 7.5% DMSO + 7.5% PROH + 0.5 mol/L sucrose in a M-199 medium with 20% SPS (20).

Oocyte vitrification was performed at room temperature (RT). The oocytes were equilibrated in ES for 5–10 min until they recovered their shape, and then they were placed into the VS for 1 min. Finally, the vitrified oocytes were placed on a CryoLoop (Hampton Research, Laguna Niguel, CA, USA) and immediately immersed in liquid nitrogen. No more than four oocytes were loaded onto each CryoLoop. Oocyte warming was performed at RT, except for the first step. The CryoLoop with the vitrified oocytes was taken out of the liquid nitrogen and immediately placed in 1.0 mol/L sucrose in a M-199 + 20% SPS solution at 37°C for 1.5–2.0 min. Next, the oocytes were placed in 0.5 mol/L sucrose in an M-199 + 20% SPS solution for 3 min at RT, after which they were transferred into another M-199 solution with 0.25 mol/L sucrose for 3 min. Finally, they were washed in M-199 + 20% SPS for 5-10 min while the stage was warmed slowly. After warming, the surviving oocytes were cultured for 2 h in G-IVF (Vitrolife, Göteborg, Sweden) in an incubator at 37°C, 6% CO_2_ before being inseminated using ICSI (20). Embryo transfer was performed on Day 2 or 3 depending on embryo quality or quantity. No more than three embryos were included in each transfer. The supernumerary embryos were cultured into blastocysts, and high-quality blastocysts were vitrified.

### Endometrial Preparation and Pregnancy Assessment

All patients used hormone replacement therapy as the endometrial preparation protocol, which has been described in a previous study (21). In short, 4–8 mg of oral estradiol valerate (Progynova, Bayer, Germany) was administered daily for at least 10 days starting on Day 2–5 of the menstrual cycle. When the endometrial thickness reached ≥8 mm, oral progesterone (Dydrogesterone, Solvay, the Netherlands) 20 mg twice daily plus vaginal micronized progesterone (Utrogestan, Besins Manufacturing Belgium) 200 mg once daily was initiated on the day of oocyte warming. Clinical pregnancy was determined as the presence of a gestational sac identified by vaginal or abdominal ultrasound 4–5 weeks after embryo transfer (ET). Gestational age, birth weight, and congenital malformation outcomes were followed-up.

### Statistical Analysis

The main outcome measurements were survival rate and the cumulative live birth rate (including live birth from fresh ETs and subsequent cryo-ETs) per warming cycle. The secondary outcome measures included laboratory outcomes of vitrified-warmed oocytes, implantation, clinical pregnancy rates, and the delivery rate per fresh embryo transfer and vitrified embryo transfer, as well as gestational age, birth weight, and congenital malformation outcomes.

The difference in means and prevalence among the groups were analyzed by Student's *t*-test for continuous data and Chi square for categorical data. A *P*-value < 0.05 was considered statistically significant. A binary logistic regression model was performed to identify predictable parameters of oocyte survival per thawing cycle. The oocyte-to-baby rate was calculated by dividing the number of live births by the total number of oocytes consumed × 100. The cumulative probability of live birth (CPLB) was estimated by the Kaplan-Meier method according to the total number of oocytes thawed in consecutive procedures, including oocytes from canceled ETs and from fresh or cryo-ETs, until a live birth was achieved.

## Results

Three hundred and twenty-one vitrified-warmed-oocyte cycles were carried out from March 2009 to October 2017 owing to unavailable sperm on oocyte retrieval day. These oocytes had previously been vitrified between 2007 and 2013. The incidence rate was around 0.3–0.5% in all IVF /ICSI cycles over the time period. The majority of cases for all-oocyte-vitrification were owing to unavailable sperm on the day from ejaculated samples or surgical sperm extraction (73.23%) (Table 1). The median age of female patients at oocyte vitrification was 30.24 years (95% CI 29.82–30.89). The median preservation duration of oocyte vitrification was 6.52 months (95% CI 5.56–7.62). Overall oocyte survival rate was 83.13% (95% CI 81.81–86.35%). Data were also obtained from age and BMI matched controls undergoing fresh ICSI cycles for severe male factor with autologous oocytes. Similar fertilization rates were shown between the vitrified and fresh groups, while the high-quality embryo rate and blastocyst rate decreased significantly in the vitrified group (Table 2).

**Table 1:** Reasons for lack of sperm availability on the day of oocyte retrieval.

**Groups**	**Cycles**	**Reasons for oocytes vitrification at fresh retrieval**
Part-oocyte-vitrified cycles	67	Insufficient sperm from ejaculated sample or surgical sperm extraction
		
All-oocyte-vitrified cycles	254	Unexpected absence of partner 23 (9.05%)
		Unable to provide ejaculated sample through masturbation 45 (17.72%)
		Unavailable sperm from ejaculated sample or surgical sperm extraction 186 (73.23%)

**Table 2:** Comparison of baseline characteristics, laboratory outcome, and clinical outcome between the oocyte vitrification group and control group.

**Groups**		**Oocytes vitrification group**	**Control group**	***P*-value**
Cycles		321	321	
Age (95% CI)		30.36 (29.82–30.89)	30.40 (29.88–30.91)	NS
BMI (95% CI)		22.97 (22.57–23.38)	23.34 (22.96–23.72)	NS
AFC		15.53 (14.73–16.32)	15.64 (14.81–16.48)	NS
Basal Hormones	FSH (IU/L) (95% CI)	6.65 (6.44–6.86)	6.64 (6.43–6.85)	NS
	LH (IU/L) (95% CI)	5.37 (5.07–5.68)	5.40 (5.07–5.73)	NS
	T0 (ng/dl) (95% CI)	26.48 (24.89–28.08)	26.72 (25.40–28.05)	NS
COH protocols	Agonist protocol	274	260	NS
	Antagonist protocol	43	54	
	Others	4	7	
Husband total progressive motile count (^*^10^6^/ml)		18.20 (11.66–24.74)	1.72 (1.30–2.14)	<0.0001
Oocytes retrieved (95% CI)		13.99 (13.29–14.69)	11.13 (10.7–11.78)	<0.0001
Oocytes warmed (95% CI)		10.25 (9.73–10.77)	–	
Preservation duration (m) (95% CI)		6.52 (5.54–7.49)	–	
Vitrified-warmed oocytes		3,290	–	
Survival oocytes/ICSI oocytes (%, 95% CI)		2,735 (84.08, 81.81–86.35)	2,992	
2PN zygotes (%, 95% CI)		1,859 (68.27, 65.65–70.89)	2,020 (68.03, 65.25–70.81)	NS
D3 high-quality embryo rate (%, 95% CI)		632 (33.33, 30.15–36.51)^#^	1,077 (53.75, 50.43–57.07)	<0.0001
Blastocyst rate per ICSI oocyte (%, 95% CI)		9.22 (7.47–10.97)	18.87(16.79–20.95)	<0.0001
Embryos transferred/cycle (95% CI)		2.08 (1.99–2.17)	1.77 (1.70–1.84)	<0.0001
Implantation rate in fresh embryo transfer cycle (%, 95% CI)		24.9 (21.2–28.6) D2/D3 transfer	42.86 (37.7–48.0) D3/D5 transfer	<0.0001
Clinical pregnancy per fresh embryo transfer cycle (%, 95% CI)		104/262 (39.69, 33.7–45.7)	118/206 (57.28, 50.5–64.1)	<0.0001
Early pregnancy loss rate in fresh embryo transfer cycles (%, 95% CI)		14/104 (13.46, 6.8–20.1)	10/118 (8.47, 3.4–13.6)	NS
Live births per transfer cycle (%, 95% CI)		109/262 (41.60, 35.6–47.6)	128/206 (62.14, 55.5–68.8)	<0.0001
Live births per frozen embryo transfer cycle (%, 95% CI)		33/81 (40.74, 29.8–51.7)	–	
Surplus vitrification blastocysts		110	627	
Congenital defect (%, 95% CI)		2^*^(1.41) (0.6–3.4)	1^#^(0.88) (0–2.3)	NS

*NS, no significance; CI, confidence interval; BMI, body mass index; AFC, antral follicle count; COH, controlled ovarian hyperstimulation; FSH, Follicle-Stimulating Hormone; LH, Luteinizing Hormone; 2PN, two pronuclei; CPLB, cumulative probability of live birth*.

* *One case of favism, one case of patent foramen ovale accompanied by ventricular septal hypertrophy;*

# *One case right polycystic kidney disease with left kidney absence, accompanied by tetralogy of Fallot*.

*^#^Cycles that had embryo transfer on D2 in the oocyte vitrification group had been excluded in D3 high-quality embryo calculations*.

Table 3 shows information from the two groups divided by median survival rate (91.67%). Serum total cholesterol in the ≥91.67% survival group was higher. The blood glucose level was also higher in the ≥91.67% survival group. There were more cycles owing to absolute male factors included in the ≥91.67% survival group. And finally, the preservation time was longer in the <91.67% survival group.

**Table 3:** Patient and cycle characteristics and CPLB in the two oocyte vitrification groups divided by the median survival rate (91.67%).

**Groups(survival rate**)		**≥91.67%**	** <91.67%**	***P*-value**
Cycles		161	160	
Age (95% CI)		30.27 (29.50–31.09) 4.92	30.44 (29.70–31.27) 4.87	NS
BMI (95% CI)		22.80 (22.19–23.35) 3.78	23.15 (22.62–23.69) 3.56	NS
TG		1.24 (1.07–1.46) 1.29	1.19 (1.04–1.34) 0.97	NS
TC		4.58 (4.42–4.75) 1.07	4.33 (4.19–4.46) 0.84	<0.05
HDL		1.34 (1.30–1.39) 0.31	1.30 (1.25–1.35) 0.34	NS
LDL		2.93 (2.81–3.08) 0.88	2.78 (2.66–2.90) 0.81	NS
Glu		5.29 (5.21–5.38) 0.56	5.15 (5.05–5.26) 0.65	<0.05
Basal Hormones	FSH (IU/L) (95% CI)	6.80 (6.51–7.12) 1.95	6.49 (6.22–6.80) 1.86	NS
	LH (IU/L) (95% CI)	5.51 (5.13–5.92) 2.57	5.24 (4.81–5.69) 2.94	NS
	T0 (ng/dl) (95% CI)	27.04 (24.78–29.49) 14.72	25.94 (23.85–28.33) 14.32	NS
COH protocols	Agonist protocol	144	130	NS
	Antagonist protocol	16	27	
	Others	1	3	
Cause of oocyte vitrification	Absolute Male factor^#^	135	118	<0.05
	Relative Male factor^*^	26	42	
Oocytes retrieved (95% CI)		13.67 (12.71–14.61) 6.17	14.31 (13.36–15.31) 6.58	NS
Preservation duration (months) (95% CI)		5.19 (4.59–5.91) 4.28	7.85 (6.27–9.75) 11.68	<0.05
Survival oocytes rate (%, 95% CI)		99.26 (98.91–99.59) 2.2	68.81, (65.56–71.81) 19.69	<0.0001
CPLB, patient (%, 95% CI)		68, 42.2 (34.5–49.9)	74,46.3 (38.4–54.1)	NS

# *Absolute male factor: Unavailable or insufficient sperm from ejaculated sample or surgical sperm*.

* *Relative male factor: Unable to provide ejaculated sample through masturbation or unexpected absence of partner*.

Table 4 gives a comparison between the two oocyte vitrification groups divided by different reasons for lack of sperm availability on oocyte retrieval day. The relative male factor group (owing to an inability to provide an ejaculated sample through masturbation or unexpected absence of partner) presented a higher serum triglyceride level. Oocyte survival rate was higher in the absolute male factor group (owing to unavailable or insufficient sperm from an ejaculated sample or surgical collection).

**Table 4:** Patient and cycle characteristics and CPLB in the two oocyte vitrification groups sorted by different reasons for lack of sperm availability on oocyte retrieval day.

**Groups (survival rate)**		**Absolute male factor^#^**	Relative male factor^*^	***P*-value**
Cycles		253	68	
Age (95% CI)		29.79 (29.22–30.42) 4.84	30.44 (29.70–31.27) 4.87	NS
BMI (95% CI)		22.83 (22.39–23.29) 3.59	23.15 (22.62–23.69) 3.56	NS
Triglyceride		1.10 (1.02–1.19) 0.71	1.6 (1.21–2.14) 2.01	<0.05
Total cholesterol		4.44 (4.31–4.56) 0.95	4.51 (4.29–4.77) 1.02	NS
High-density lipoprotein		1.33 (1.29–1.37) 0.33	1.28 (1.21–1.35) 0.29	NS
Low-density lipoprotein		2.83 (2.72–2.95) 0.86	2.96 (2.77–3.16) 0.79	NS
Blood glucose		5.20 (5.11–5.28) 0.61	5.32 (5.19–5.47) 0.59	NS
Basal hormones	FSH (IU/L) (95% CI)	6.63 (6.40–6.84) 1.77	6.81 (6.26–7.42) 2.29	NS
	LH (IU/L) (95% CI)	5.33 (5.03–5.62) 2.45	5.64 (4.90–6.56) 3.68	NS
	T0 (ng/dl) (95% CI)	26.95 (25.09–28.79) 14.59	24.75 (21.69–28.25) 14.21	NS
COH protocols	Agonist protocol	217	57	NS
	Antagonist protocol	35	8	
	others	1	3	
Oocytes retrieved (95% CI)		14.05 (13.29–14.80) 6.40	13.78 (12.31–15.29) 6.31	NS
Husband total progressive motile count (^*^10^6^/ml)		8.67 (4.08–13.26)	72.62 (42.87–102.42)	<0.0001
Preservation duration (months) (95% CI)		6.88 (5.79–8.05) 9.43	5.15 (3.92–6.74) 6.24	NS
Survival oocytes rate (%, 95% CI)		85.76 (83.24–88.18) 19.70	77.85 (72.20–82.94) 23.05	<0.0001
CPLB, patient (%, 95% CI)		112, 44.3 (38.1–50.4)	30, 44.1 (32.0–56.2)	NS

# *Absolute male factor: Unavailable or insufficient sperm from ejaculated sample or surgical sperm*.

* *Relative male factor: Unable to provide ejaculated sample through masturbation or unexpected absence of partner*.

Table 5 shows the comparison of sibling oocytes between fresh and vitrified groups in part-oocyte-vitrified cycles. A total of 67 cycles had a portion of oocytes vitrified because of male factors. Forty-one cases were inseminated with the husband's sperm in both fresh oocytes and vitrified oocytes. No significant difference was found between the vitrification and fresh groups of sibling oocytes in fertilization rate (66.93 vs. 59.77%), but fewer high-quality embryos developed in the vitrified group (27.68 vs. 53.46%).

**Table 5:** Sibling oocytes compared in part-oocyte-vitrified cycles (survival of oocytes inseminated with sperm from the husband).

**Cycles**	**41**		
**Groups**	**Fresh oocytes**	**Vitrified-warmed Oocytes**	***P*-value**
Number of oocytes	233	338	–
Survival oocytes (%, 95% CI)	–	299 (90.24, 84.20–96.28)	–
2PN zygotes (%, 95% CI)	143 (59.77, 48.63–70.91)	207 (66.93, 57.99–75.88)	NS
D2 transfer cycles (No. embryos)	10 (16)	9 (23)	–
D3 transfer cycles (No. embryos)	18 (28)	20 (41)	–
D3 high-quality embryos^*^ (%, 95% CI)	53/126 (53.46, 39.05–67.86)	53/177 (27.68, 18.34–37.02)	<0.0001
Live birth per oocyte retrieval cycle or warmed cycle (%, 95% CI)	4/41 (9.5, 3.0–18.8)	17/41 (40.48, 25.0–56.0)	<0.0001

* *Cycles that had embryos transfer on D2 in the oocyte vitrification group were excluded in D3 high-quality embryos calculations*.

Table 6 shows the clinical outcomes according to different sperm sources after oocyte warming in all-oocyte-vitrified cycles. Among 254 cycles, the warmed oocytes were fertilized with the husbands' sperm, testicular sperm aspiration/percutaneous epididymal sperm aspiration (PESA/TESA) sperm, and frozen donor sperm in 150, 46, and 58 cycles, respectively. The frozen donor sperm group showed a better high-quality-embryo rate than the husbands' sperm and the PESA/TESA sperm group (40.94 vs. 32.76%; 40.94 vs. 31.95%).

**Table 6:** Clinical outcomes according to different sperm sources after oocyte warming in all-oocyte-vitrified cycle groups.

	**All oocytes vitrified cycles (254 cycles)**		
	**Husband semen**	**Husband PESA/TESA sperm**	**Donor frozen sperm**
	150	46	58
Age	31.73 (30.93–32.53)	29.48 (28.16–30.80)	30.26 (28.97–31.55)
Number of oocytes	1,576	474	664
Oocyte survival (%, 95% CI)	1,288 (81.67, 78.19–85.16)	396 (86.17, 79.69–92.65)	557 (84.27, 79.22–89.32)
Fertilized oocytes (%, 95% CI)	873 (68.19, 64.08–72.31)	266 (69.70, 62.68–76.72)	381 (67.92, 63.23–72.62)
D2 fresh transfer cycles (No. embryos)	17 (37)	5 (10)	6 (13)
D3 fresh transfer cycles (No. embryos)	90 (191)	29 (62)	46 (94)
D3 high quality embryos (%, 95% CI)	246 (33.38, 27.79–38.97)^a^	81 (31.01, 23.40-−40.24)^a^	148 (41.89, 35.60–48.30%)^b^
Cumulative live birth per warmed cycle (%), (95% CI)	36.0, (28.2–43.8)^a^	50.0, (35–65)^a^	74.14, (62.5–85.8)^b^

*Different superscripts in the same row indicate statistical differences (P < 0.05)*.

There were 81 vitrified embryo transfer cycles, including 53 double frozen transfers (i.e., vitrified oocyte and vitrified embryo) cycles and 28 triple frozen (i.e., vitrified oocyte, frozen sperm, and vitrified embryo) transfer cycles, which yielded 22 and 11 neonates, respectively. The delivery rate per transfer, gestational age, birth weight, and congenital malformation outcomes were similar among groups.

**Figure 2:**
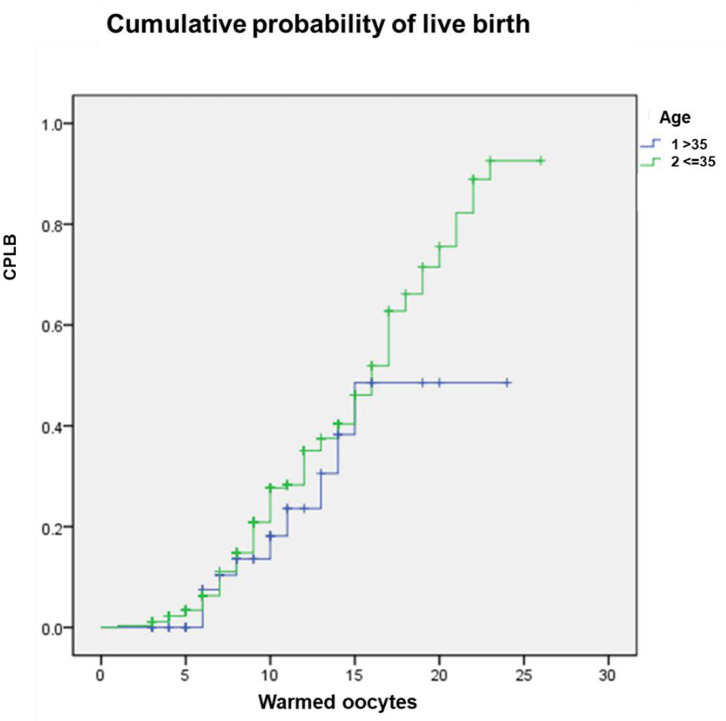
The cumulative probability of live birth according to age (≤35 vs. >35 year) and number of oocytes thawed.

One hundred and forty-two babies were born as a result of 262 fresh ETs and 81 subsequent cryo-ETs. The cumulative live birth rate per warming cycle was 41.40%. The oocyte-to-baby rate was 4.3%. At the end of the present study, 110 blastocysts remained cryopreserved from the oocyte warming cycles included in this work. Assuming that the delivery rates are maintained with this cohort, a rough estimation after their use could yield an outcome of 36 additional babies, which would enhance the oocyte-to-baby rate to 5.4%. The Kaplan-Meier analysis showed no significantly different CPLB between patients ≤35 vs. >35 years (Log-rank (Mantel-Cox); *P* = 0.231; Breslow (generalized-Wilcoxon); *P* = 0.458; and Tarone–Ware; *P* = 0.388). The CPLB improved when more oocytes were warmed and the curve for older patients reached a plateau earlier (with 15 oocytes) than those for young women (with 23 oocytes) (Figure 2).

A binary logistic regression model was performed to find predictable parameters of oocyte survival per thawing cycle. Several parameters were introduced into the initial model as predictors, including age, BMI, metabolic indicators (including triglyceride, total cholesterol, high density lipoprotei, etc.), basic hormones, infertility years, polycystic ovary syndrome (PCOS)/non-PCOS, endometriosis/non-endometriosis, ovarian stimulation protocols, reason for lack of sperm availability, vitrification kits, and storage duration. As shown by the odds ratio (OR), the effect of reason for lack of sperm availability was acknowledged, and the effect of serum TC on survival was suggested (Supplementary Table 2).

## Discussion

Given that oocyte cryopreservation techniques have changed from slow freezing to vitrification according to the safety and efficacy of reports over the past decade (22), oocyte vitrification has been gradually introduced into assisted reproduction treatment in various clinical scenarios. In particular, oocyte vitrification is becoming an indispensable alternative technique for couples who do not have sufficient available sperm at the time of egg retrieval (18). Our study represents the findings of the largest data set from a single center in China of vitrified autologous oocytes, which were obtained from couples who lacked available sperm at the time of egg retrieval. This report comprises 321 oocyte-vitrification-warming cycles. A total of 142 healthy babies born from fresh and frozen embryo transfer and the cumulative live birth rate per warming cycle was 44.24%. We found that oocyte survival was better in those couples with absolute male factors.

Different oocyte sources, including cancer patients, women desiring fertility preservation, oocyte donors, or infertile patients, may exist with different inherent qualities that influence vitrification outcomes (3, 18, 21, 23). The current study added more information, which is not optimistic, regarding oocyte vitrification in infertile patients. Inconsistent with previous reports regarding recipient oocyte vitrification cycles (13, 17, 24), in the present study, the high-quality embryo rate and blastocyst rate in vitrified oocytes decreased significantly compared with fresh oocytes (Table 2). Similar outcomes were found in the sibling oocyte comparison from part-oocyte-vitrified cycles (Table 5). Further evidence in the present study was from the comparison between groups with different reasons for the lack of sperm availability. Survival rate was significantly higher in the absolute male factor group; this may be because the women in this group were relatively “fertile” and might have had higher quality oocytes (Table 4). In the all-oocyte-vitrified cycles, which were divided into three groups depending on the sperm resource used after oocyte warming, the donor sperm group showed better embryo quality compared with the husband sperm groups. The explanation here may be that both oocytes and sperm in this group were from relatively “fertile” individuals (Table 6). Literature also reports similar results; that vitrification could damage oocyte potential from infertile women in egg-sharing or autologous oocyte vitrification programs (25, 26). All these outcomes demonstrate that oocytes from infertile women are more vulnerable to vitrification injury and might not easily survive the vitrification-warming procedure.

In order to obtain more referential information for clinical work, we tried to find some useful predictors of successful outcome following oocyte vitrification. Age was firstly taken into consideration. However, in the present study, no significant difference was discovered between the two age groups (≤35 vs. >35 years) in survival rate, fertilization rate, and high-quality embryo rate (Supplementary Table 1). These results were confirmed by the Kaplan-Meier analysis of CPLB according to different age groups. No significant difference was observed between the two age groups (Figure 1). This result was inconsistent with previous studies (7, 9, 13, 17), most probably owing to the small sample size and characteristics of the older patients involved in the present work. Only 50 (15.68%) patients >35 years old were included, because most advanced age couples are more inclined to choose donor sperm in cases of unavailable sperm on oocyte retrieval day. The average number of retrieved oocytes in this older age group was 11.46 (95% CI 9.70–13.22), which indicated a better ovarian reserve of these patients than their peers and further explained the insignificant difference between the two age groups. However, we observed that the older patients' curve reached a plateau earlier than for younger women, which agreed with other studies (9, 13, 17).

The average survival rate in the present study was 83.13% (95% CI 81.81–86.35%), comparable to the published data range from 68.6 to 96.8% (12, 26–29) in the literature. We compared two groups divided by the median survival rate (91.67%). Statistical differences were found in the serum total cholesterol, the proportion of different reasons for lack of sperm availability, and preservation time. The outcomes were partially consistent with multiple logistic regression analysis. As shown by the adjusted OR, the effect of the reason for lack of sperm availability was reassuring. Another parameter entered in the model was serum TC, which had not previously been analyzed in human oocyte vitrification studies. A higher serum TC level was found to be favorable for oocyte survivability after vitrification. Cholesterol is known to be the major non-polar lipid in mammalian cell membranes (30). Modulation of plasma membrane cholesterol to increase post-cryopreservation survival is currently a new topic in mammalian oocyte vitrification (31–33). Large prospective research studies are needed to confirm whether serum lipid levels or other metabolic parameters, are relevant to oocyte survivability after vitrification.

Oocyte vitrification efficiency could be defined as the route to a live birth using the lowest number of vitrified oocytes. Although we have obtained a cumulative live birth rate per warming cycle of 44.24%, the oocyte-to-baby rate was only 4.3% in the present study. About one third of couples (36.76%) had successfully taken babies home. Other studies addressing oocyte vitrification for medical indications have reported quite different outcomes of oocyte-to-baby rate. Kara et al. reported the live-birth rate per mature oocyte was 3.0% in an oocyte cryopreservation group (<35 years old) (25). Doyle et al. estimated live birth per warmed oocyte as 6.5% (including predicted live birth from remaining cryopreserved blastocysts) (12). The data herein provides more information for clinicians to advise patients faced with the situation of unavailable sperm on oocyte retrieval day.

The outcomes of live delivery, including gestational age, birth weight, and live birth congenital defects were compared with the fresh control group, and no significant differences were noted. The limited data we collected showed that double vitrification (oocyte and embryo vitrification) or triple-cryopreservation (oocyte/embryo vitrification and sperm cryopreservation) had no adverse effects on perinatal outcomes.

The population in our study was very special and it is impossible to carry out a prospectively randomized controlled trial in such situations for ethical and legal reasons. The long duration of this retrospective study might have added some variations that may have affected the presented data. However, we have a relatively stable laboratory team with experienced technicians trained in oocyte vitrification. Furthermore, we included stimulation protocols and vitrification kit parameters that changed through time in the regression model as potential confounders. Another drawback was the relatively limited sample size, for the incidence rate was only 0.3–0.5% in all IVF/ICSI cycles during data collection years in our hospital. Finally, the couples in the present study mostly exhibited severe male factors, which could influence subsequent embryo development and pregnancy outcomes. Therefore, the outcome results might not represent the entire medical indications for oocyte preservation.

## Conclusions and Perspectives

Oocyte vitrification is an indispensable and effective alternative when there is a lack of available sperm on oocyte retrieval day. The present study showed that oocyte survival was better in couples with absolute male factors and this suggested that oocytes from infertile women were more likely to suffer from vitrification injury. Further studies will be necessary to clarify the correlation between serum metabolism parameters and human oocyte survival after vitrification. Our study has preliminarily contributed to the important question for clinical practice of how to distinguish the female population who have oocytes with better survivability after vitrification. We hope more data from autologous oocyte vitrification studies with a large-scale and controlled variable design could add to and clarify our results.

## Data Availability Statement

The original contributions presented in the study are included in the article/Supplementary Material, further inquiries can be directed to the corresponding author/s.

## Ethics Statement

Written informed consent was obtained from the individual(s) for the publication of any potentially identifiable images or data included in this article.

## Author Contributions

SG and JM contributed to the study concept and design of this study. XF analyzed data and drafted the paper. XL, JL, and MZ contributed to the review and the revision of the manuscript. All authors approved the final submitted and published versions.

## Funding

This study was supported by the National Key Research and Development Program of China (2016YFC1000202 and 2018YFC1002804).

## Conflict of Interest

The authors declare that the research was conducted in the absence of any commercial or financial relationships that could be construed as a potential conflict of interest.

## Publisher's Note

All claims expressed in this article are solely those of the authors and do not necessarily represent those of their affiliated organizations, or those of the publisher, the editors and the reviewers. Any product that may be evaluated in this article, or claim that may be made by its manufacturer, is not guaranteed or endorsed by the publisher.
