# Supplementary material for: An Eight Year Experience of Autologous Oocyte Vitrification for Infertile Patients Owing to Unavailability of Sperm on Oocyte Retrieval Day
Source: Front Med (Lausanne). 2021 Oct 26;8:663287. doi: 10.3389/fmed.2021.663287 (PMC8575774; doi:10.3389/fmed.2021.663287)
Supplement: Supplementary file 1 [file Table_1.DOCX]

Supplementary Table I Laboratory outcome and clinical outcome according to the patients’ age at vitrification

| Groups | | ≤35 years | >35 years | P value |
| --- | --- | --- | --- | --- |
| Cycles | | 271 | 50 |  |
| Age (95% CI) | | 28.83 (28.41-29.25) | 38.62 (37.90-39.34) | <0.0001 |
| BMI (95% CI) | | 22.73 (22.31-23.14） | 24.28 (23.03-25.53) | 0.016 |
| Basal Hormones | FSH(IU/L) (95% CI) | 6.51 (6.31-6.71) | 7.38 (6.57-8.19) | 0.041 |
|  | LH(IU/L) (95% CI) | 5.45 (5.12-5.78) | 4.94 (4.17-5.72) | NS |
|  | T0(ng/dl) (95% CI) | 27.31 (25.62-29.02) | 21.86 (17.41-26.31) | 0.015 |
| COH protocols | Agonist protocol | 230 | 44 | NS |
|  | Antagonist protocol | 39 | 4 |  |
|  | others | 2 | 2 |  |
| Oocytes retrieved (95% CI) | | 14.46 (13.70-15.21) | 11.46 (9.70-13.22) | 0.02 |
| Oocytes warmed (95% CI) | | 10.46（9.91-11.03） | 9.06 (7.74-10.39) | NS |
| Preservation duration (d) (95% CI) | | 196.61 (165.35-227.86) | 189.72 (106.34-273.10) | NS |
| Vitrified-warmed oocytes | | 2837 | 453 |  |
| Survival oocytes (%, 95% CI) | | 2350 (83.68, 81.17-86.19) | 385 (86.28, 80.89-91.67) | NS |
| 2PN zygotes (%, 95% CI) | | 1601 (67.98, 65.13-70.83) | 258 (68.49, 61.21-75.76) | NS |
| D3 high-quality embryo rate (%, 95% CI) | | 549/1601 (33.11, 29.71-36.51） | 83/258 (35.18, 26.05-44.32) | NS |
| Embryos transferred/cycle (95% CI) | | 2.04 (1.97-2.12) | 2.00 (1.78-2.21） | NS |
| Implantation rate in fresh embryo transfer cycle (%, 95% CI) | | 119/452 (26.32, 22.3-30.4) | 6/82 (7.31, 1.6-13.1) | <0.0001 |
| Clinical pregnancy per fresh embryo transfer cycle (%, 95% CI) | | 97/221 (43.89, 37.3-50.5) | 6/41 (14.63, 3.3-25.9) | <0.0001 |
| Early pregnancy loss in fresh embryo transfer cycles (%, 95% CI) | | 14/97 (14.43, 7.3-21.6) | 0/6 |  |
| CPLB/patient (%, 95% CI) | | 132/271 (48.71, 42.7-54.7) | 10/50 (20, 8.5-31.5) | <0.0001 |

Supplementary Table II Binary Logistic regression (BLR) models for oocyte survival considering considering patients and cycles parameters. TC(total cholesterol), the reason for lack of sperm availability and preservation time were entered into the equation.

| Confounders | Adj. OR | CI95% | P value |
| --- | --- | --- | --- |
| TC | 1.409 | 1.099-1.823 | 0.009 |
| reason for lack of sperm availability | 1.960 | 1.105-3.474 | 0.021 |
| preservation time | 0.955 | 0.919-0.993 | 0.021 |

Supplementary Table III Clinical outcomes according to different sperm sources after oocytes warming in all-oocyte-vitrified cycles groups

|  | All-oocyte-vitrified cycles (254 cycles) | | |
| --- | --- | --- | --- |
| Groups | Husband semen | Husband PESA/TESA sperm | Donor frozen sperm |
| Cycles | 150 | 46 | 58 |
| Age (95% CI) | 31.73 (30.93-32.53) | 29.48 (28.16-30.80) | 30.26 (28.97-31.55) |
| Number of oocytes | 1576 | 474 | 664 |
| Survival oocytes (%, 95% CI) | 1288 (81.67, 78.19-85.16) | 396 (86.17, 79.69-92.65) | 557 (84.27, 79.22-89.32) |
| 2PN zygotes (%, 95% CI) | 873 (68.19, 64.08-72.31) | 266 (69.70, 62.68-76.72) | 381 (67.92, 63.23-72.62) |
| D2 fresh transfer cycles (No. embryos) | 17(37) | 5(10) | 6（13） |
| D3 fresh transfer cycles (No. embryos) | 90(191) | 29(62) | 46（94） |
| D3 high-quality embryos*(%, 95% CI) | 246 (33.38, 27.79-38.97) a | 81 (31.01, 23.40-40.24) a | 148 (41.89, 35.60-48.30) b |
| Cumulative live birth per warmed cycle (%, 95% CI) | 54/150 (36.0, 28.2-43.8) a | 23/46 (50.0, 35-65) a | 43/58 (74.14, 62.5-85.8) b |

Note: Different superscripts in the same row indicate statistical differences (P<.05).

* Cycles had embryos transfer on D2 had been excluded in D3 high-quality embryos calculation.

Supplementary Table IV Clinical outcome and perinatal outcome according to the different series of cryopreservation.

|  | Fresh embryo transfer in vitrified-warmed cycle | DF* transfer cycles | TF# transfer cycles | Control group fresh transfer |
| --- | --- | --- | --- | --- |
| Transfer cycles | 262 | 53 | 28 | 206 |
| Clinical pregnancy per transfer cycle (%, 95% CI) | 104/262(39.69, 33.7-45.7) a | 29/53(54.71, 40.9-68.6) b | 14/28 (50, 30.3-69.7) ab | 118/206 (57.28, 50.5-64.1)b |
| Delivery per transfer cycle (%, 95% CI) | 89/262(33.97, 28.2-39.7) a | 21/53 (39.62, 26.0-53.2) b | 10/28 (35.71, 16.8-54.6) ab | 96/206 (46.60, 39.7-53.5) b |
| Live birth per transfer cycle (%, 95% CI) | 109/262 (41.60,35.6-47.6) a | 22/53 (41.51, 27.8-55.2) a | 11/28 (39.29, 20.0-58.6) a | 128/206 (62.14, 55.5-68.8) b |
| Single delivery (%, 95% CI) | 69 (77.53, 68.7-86.4) | 20（95.24） | 9（90） | 68（70.83）（61.6-80.1） |
| Median gestational age (weeks) singleton pregnancies (95% CI) | 274.91(271.58-278.25) | 283.45(272.13-294.77) | 276.00(268.71-283.29) | 272.99(269.50-276.47) |
| Median gestational weight (gm) singleton pregnancies (95% CI) | 3.48 (3.35-3.60) | 3.50 (3.35-3.65) | 3.14(2.77-3.50) | 3.41(3.29-3.53) |
| Twin delivery | 20 | 1 | 1 | 30 |
| Median gestational age (days) singleton pregnancies (95% CI) | 256.20(250.23-262.17) | -- | -- | 254.83(247.99-261.68) |
| Median birth weight (Kg) singleton pregnancies (95% CI) | 2.54(2.39-2.69) | -- | -- | 2.52(2.36-2.67) |
| Congenital defect | 2 | 0 | 0 | 1 |

Note: Different superscripts in the same row indicate statistical differences (P<0.05).

*DF transfer: vitrified oocyte and vitrified embryo.

#TF transfer: vitrified oocyte, frozen sperm and vitrified embryo.
